# Switching to the CKD-EPI but Not Modified FAS eGFR Formula Underdetects CKD Among Adolescents and Young Adults in México

**DOI:** 10.3390/children12020239

**Published:** 2025-02-17

**Authors:** Alethia Paulina Monserrat Guzmán Núñez, Guido Filler, Olivier C. Barbier, Elodia Rojas Lima, Pablo Mendez-Hernández, Manolo Ortega-Romero, Maria Esther Díaz González de Ferris, Mara Medeiros

**Affiliations:** 1 Department of Paediatrics, Hospital Infantil de México Federico Gómez, Mexico City 06720, Mexico; paulina_gn7@hotmail.com; 2 Department of Paediatrics, Western University, London, ON N6A 3K7, Canada; 3 Department of Medicine, Western University, London, ON N6A 3K7, Canada; 4 The Lilibeth Caberto Kidney Clinical Research Unit, Western University, London, ON N6A 3K7, Canada; 5 Children’s Health Research Institute, London, ON N6C 4V3, Canada; 6 Departamento de Toxicología, Centro de Investigación y de Estudios Avanzados del Instituto Politécnico Nacional, Mexico City 07360, Mexico; obarbier@cinvestav.mx (O.C.B.); elodia.rojas@secihti.mx (E.R.L.); 7 Jefatura de Educación, Investigación y Capacitación del Hospital General Tlaxcala, Secretaría de Salud de Tlaxcala, Santa Ana Chiautempan 90800, Mexico; pmendezh@uatx.mx; 8 Facultad de Ciencias de la Salud, Universidad Autónoma de Tlaxcala, Tlaxcala de Xicohténcatl 90000, Mexico; 9 Unidad de Investigación en Nefrología y Metabolismo Mineral Óseo, Hospital Infantil de México Federico Gómez, Mexico City 06720, Mexico; manolo.ortega@secihti.mx (M.O.-R.); mmedeiros@himfg.edu.mx (M.M.); 10 Department of Pediatrics, The University of North Carolina at Chapel Hill, Chapel Hill, NC 27599, USA; maria_ferris@med.unc.edu; 11 Departamento de Farmacología, Facultad de Medicina, Universidad Nacional Autónoma de México, Mexico City 04510, Mexico

**Keywords:** CKiD U25 formula, Pottel Full Age Spectrum (FAS) formula, modified Schwartz formula, CKD-Epi formula, serum creatinine, at-risk population, adolescents and young adults (AYA) population, kidney disease of unknow origin (KDu)

## Abstract

Background: Guidelines recommend switching the glomerular filtration rate (eGFR) estimation from the CKiD-U25 to the CKD-EPI formula at age 18. We investigated how this would affect chronic kidney disease (CKD) classification. Methods: Serum creatinine was enzymatically measured in 1061 samples from 914 community-based 10–23-year-olds from Tlaxcala, Mexico, a region where urinary biomarkers demonstrated early kidney damage associated with exposure to inorganic toxins in a pediatric population. We calculated their eGFR using CKiD-U25, modified Schwartz, the first and modified Pottel full-age spectrum (FAS), and CKD-EPI formulae. Correlation analysis characterized the CKD stage stratified by age and sex. Results: At baseline, the median age was 13 (IQR: 12, 15) years, and 55% were female. Median CKiD-U25 eGFR was 96.9 (IQR: 83.3, 113.3) mL/min/1.73 m^2^, significantly lower than the CKD-EPI eGFR, which was 140.8 (IQR: 129.9, 149.3) mL/min/1.73 m^2^ (*p* < 0.0001, Wilcoxon rank test). The mean bias was 36.99 ± 12.89 mL/min/1.73 m^2^. Pearson correlation was r = 0.8296 (95% confidence interval 0.0898–0.8474). There was a better correlation between the modified Schwartz (r = 0.9421 (0.9349, 0.9485)) and the Pottel FAS (r = 0.9299 (0.9212, 0.9376)) formulae. Agreement was deficient when the eGFR was >75 mL/min/1.73 m^2^ in younger age and female sex. Modified Schwartz identified 281 (26.4%) measurements as having CKD 2 and 3 (2+), U25 identified 401 (37.7%) measurements as having CKD 2+, FAS identified 267 (25.1%) and modified FAS identified 282 (30%) measurements as having CKD 2+, and CKD-EPI identified 51 (4.8%) measurements as having CKD 2+, respectively. Conclusions: In this population, there needed to be better agreement between the various eGFR formulae. CKD-EPI identifies substantially fewer at-risk participants as having CKD.

## 1. Introduction

Over the life cycle, nephron endowment is finite at 34–36 weeks’ gestation [1]. Kidney disease and nephron loss during childhood may be associated with an increased risk of end-stage kidney disease (ESKD) when patients are in their thirties [2,3,4]. Since there are effective measures to slow the progression of chronic kidney disease (CKD), such as blockage of the renin–angiotensin–aldosterone system [5] and strict blood pressure control [6], early identification of CKD is paramount [7].

We recently described a community-based cohort of 109 healthy pre-adolescents, adolescents, and young adults from Apizaco, Tlaxcala, México, who were exposed to inorganic toxins, and discovered a high proportion of CKD using enzymatically determined serum creatinine [8]. Hereafter, we will refer to adolescents and young adults as ‘AYA’ throughout the manuscript. This region of México belongs to Meso-America, an area at risk for kidney disease of unknown origin (KDu) [8,9]. The estimated glomerular filtration rate (eGFR) was calculated using the modified Schwartz formula [10]. We have augmented that original sample of healthy AYA to 914 individuals from Tlaxcala and Apizaco. We followed updated guidelines for GFR estimation, using the Pierce CKiD U25 formula [11] (from here on referred to as U25), primarily derived from patients with CKD, with enzymatically determined serum creatinine as recommended for individuals aged 2–25 years. As per the Kidney Disease: Improving Global Outcomes (KDIGO) guidelines [12], we switched to CKD-EPI at age 18 [13] and noticed a substantial increase in eGFR. This increase is similar to a study conducted by Nyman et al. [14]. Webster-Clark et al. demonstrated that creatinine-based eGFR using modified Schwartz and CKD-EPI doesn’t converge until age 25–30 [15]. Selistre has also reported that continuous use of pediatric formulae until age 40 may be more appropriate [16]. Pottel et al. argued similarly with their full age spectrum (FAS) eGFR equation. More recently, a modified FAS equation was published [17]. The level of eGFR increase when switching from pediatric to adult formulae (per current guidelines) is implausible. However, while the lack of agreement has been subject to a few publications, the degree of overestimation and the impact on re-classifying CKD stages has been understudied.

We compared the identification of glomerular hyperfiltration, which is characterized by an increase in the filtration rate of individual nephrons, known as single-nephron glomerular filtration rate (SNGFR). Since direct measurement of SNGFR is not feasible in clinical practice, whole-kidney GFR is often used as a surrogate. Elevated SNGFR can indicate a compensatory response to nephron loss or an early pathological state associated with kidney disease progression. Most authors agree that an eGFR of >135 mL/min/1.73 m^2^ is a reasonable upper-level threshold. Correct identification of the CKD stage is essential to identifying patients at risk. We recently described that the diagnostic accuracy of the U25 and the CKD-EPI against measured GFR (mGFR) using a high-quality ^99^TcDTPA clearance method in 2–20-year-old Canadian patients could perform better, especially when the mGFR is >75 mL/min/1.73 m^2^, even when combining the estimation with cystatin C and creatinine [18]. Although serum creatinine is widely used to estimate GFR, it has inherent limitations and biases. Creatinine levels are influenced by factors such as muscle mass, age, sex, hydration status, and dietary protein intake, which can lead to overestimation or underestimation of kidney function. Additionally, tubular secretion of creatinine and variability in laboratory assays contribute to inconsistencies in GFR estimation, particularly in young adults transitioning from pediatric to adult eGFR formulas. Serum creatinine is the only biomarker of GFR in many less-resourced countries.

In a community-based Mexican cohort of apparently healthy AYA from the State of Tlaxcala, we estimated GFR using the modified Schwartz formula [10] (externally validated), the U25 formula by Christopher Pierce [11], the Full Age Spectrum (from hereon named FAS) formula by Hans Pottel (externally validated) [19], the modified FAS formula, and the CKD-EPI formula by Leslie Inker (which included only a few young adults) [13]. All of these formulae were used for targeted screening for CKD. We hypothesized that switching from pediatric formulae to CKD-EPI would change the CKD classification. Our main objective was to identify how different the CKD stage classifications would be and how many at-risk patients would not be identified as having CKD when using the CKD-EPI formula [20].

## 2. Materials and Methods

### 2.1. The Setting, Participants, Ethics, and Funding

We conducted the study in a community-based cross-sectional study of 914 consecutive seemingly healthy 10 to 23-year-old persons from Tlaxcala, Chiautempan, and Apetatitlán, three municipalities from the state of Tlaxcala, México, a Meso-American area where the level of environmental toxins could place people at-risk for CKD [8]. A small group of patients agreed to lifestyle intervention and repeated creatinine measurements. All other patients only had one measurement.

Exclusion criteria included a history of renal disease, fever 48 h before the study, physical activity for >2 h the day before sampling, and, for girls, being on their menses. Samples were collected from September 2019 to September 2023.

We calculated the sample size for a cross-sectional study, considering a 95% confidence interval (CI), a precision of 5%, and an expected prevalence of 0.31% (Tlaxcala State Registry of CKD cases, 2016). The study was conducted in full compliance with the Declaration of Helsinki. The ethics committees of the Hospital Infantil de México Federico Gómez (HIMFG), Secretaría de Salud de Tlaxcala, and the Institutional Bioethics Committee for Research in Humans (COBISH-Cinvestav) approved the study. This study was funded by the Consejo Nacional de Ciencias y Tecnología (CONACYT), a Mexican government agency, through the HIMFG in Mexico City.

### 2.2. Data Collection

Data were collected from participants and caregivers (who signed informed consent forms for minors) before enrolling in the study. Participants underwent a medical exam with duplicate measurements of manual blood pressure using a sphygmomanometer; weight was determined with a clinical scale and height with a stadiometer. Body mass index (BMI) was calculated using the STAT Growth Charts TM application version 3.2 from the Centers for Disease Control. Body mass index (BMI) categories were defined according to the World Health Organization (WHO) growth standards: low weight (<5th percentile for age and sex), normal weight (5th–85th percentile), overweight (85th–95th percentile), and obesity (>95th percentile), as per the same reference. The administered questionnaires collected socio-environmental information, including household characteristics, personal/family health history of chronic-degenerative conditions, and environmental exposures. Poverty classification followed the criteria established by the Mexican National Council for the Evaluation of Social Development Policy (CONEVAL) available at https://www.coneval.org.mx/. Moderate poverty included households with limited access to essential services such as education, healthcare, and adequate nutrition, while extreme poverty was defined as the inability to meet basic food and non-food needs, indicating severe deprivation of essential living conditions.

Microalbumin and multistix strips were tested immediately since the urine results were given to the participants the same day (approximately one hour after receiving them). We added this information to the methods section. Peripheral venipunctures obtained blood samples (in some cases longitudinally). Serum samples were collected in serum separator tubes and centrifuged at the site at 3500× *g* for 10 min. The samples were maintained at 4° centigrade after centrifugation and aliquoted. The samples were shipped to the Federico Gomez Children’s Hospital between 6 to 8 h post-harvest, keeping constant care of the cold chain. Serum creatinine was measured in a central reference laboratory at HIMFG using the DIMENSION RxL Max SIEMENS equipment (Siemens Healthineers, Erlankgen, Germany) and the enzymatic method, and it was traceable by non-isotope-dilution mass spectrometry. The accuracy and consistency of the creatinine results were frequently assessed according to standard laboratory procedures. The intra-assay performance of the creatinine assay using human samples at 66.5 ± 2.8 µmol/L had a coefficient of variation of 4.0%, and, at 548 ± 5 µmol/L, it had a coefficient of variation of 0.8%. The inter-assay performance at 56.0 ± 3.1 µmol/L had a coefficient of variation of 5.5%, and, at 584 ± 8 µmol/L, it had a coefficient of variation of 1.4%. eGFR was calculated using the modified Schwartz [10], the U25 [11], the FAS [19], and the CKD-EPI formulae [13]. The modified FAS formula was only used for the subgroup analysis.

The eGFR formulas included in our study were derived from different populations with distinct clinical characteristics:

- **CKD-EPI** (Levey et al. [13]) was developed primarily using data from adults, including a mix of healthy individuals and CKD patients, making it the standard for estimating GFR in adults but potentially less accurate in younger populations.
- **Modified Schwartz** (Schwartz et al. [10]) was derived from children and adolescents with CKD and remains the gold standard for pediatric GFR estimation.
- **U25** (Pierce et al. [11]) was specifically designed for individuals aged 2–25 years with CKD, incorporating enzymatic creatinine measurements to improve accuracy in this age group.
- **Full Age Spectrum (FAS)** (Pottel et al. [19]) was developed using data spanning all age groups, including both healthy and CKD populations, allowing for continuity from childhood to adulthood.
- **Modified FAS** (Pottel et al. [17]) was an update to the original FAS equation, incorporating refinements to better account for variations in creatinine production across ages.

By including both pediatric and adult-derived equations, our study aimed to highlight the impact of transitioning from pediatric to adult eGFR estimation methods and the potential misclassification of CKD risk in adolescents and young adults.

### 2.3. Statistics

We used the D’Agostino Pearson Omnibus test to assess the sample’s normality of the distribution and used parametric or non-parametric descriptive statistics, as appropriate. We compared the various estimates using Pearson correlation and plotted the four formulae against age and stratified by sex. A *p*-value < 0.05 was considered significant. The agreement was analyzed by plotting matched eGFRs against age. We performed a subgroup analysis for hyperfiltration using a cut-off of >135 mL/min/1.73 m^2^. We also calculated the proportion of participants whose eGFR was >90 mL/min/1.73 m^2^ (CKD stage 1), >60–89 mL/min/1.73 m^2^ (CKD stage 2), and >30–59 mL/min/1.73 m^2^ (CKD stage 3). There were no patients with CKD stages 4 or 5. We compared the proportions using the Chi-square test and used analysis of variance (ANOVA) with Geisser–Greenhouse correction to compare differences between means. When comparing medians, we used non-parametric ANOVA with Friedman statistics.

## 3. Results

### 3.1. Study Population

Table 1 summarizes the baseline characteristics of the 914 study participants aged 10–23. Including the 121 participants who had a second assessment at one year and the 27 who had a third assessment at two years, we had 1061 eGFR not normally distributed. Twenty-eight patients had measurements two years apart.

### 3.2. Analysis of All GFR Estimations

**Figure 1:**
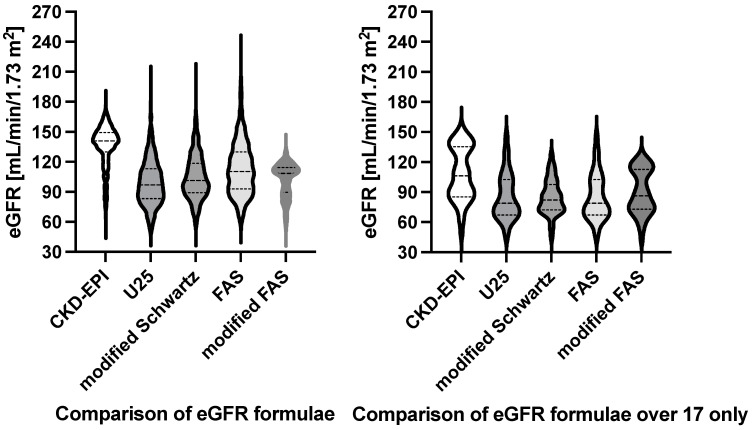
Violin plot of the four GFR estimates. This figure has two panels. The left panel regards the entire patient group, and the right panel is for patients over 18 only. CKD-EPI—Inker’s eGFR formula for adults [13]; U25—Pierce’s CKiD eGFR for patients aged 2–25 years of age [11]; modified Schwartz—Schwartz’ updated formula of the bedside eGFR for enzymatic creatinine measurements [10]; FAS—Pottel’s full age spectrum [19]; modified FAS—modified Pottel’s full age spectrum [17].

The four eGFR formulae calculations yielded very different results, summarized in Table 2. All eGFRs were significantly different (*p* < 0.0001, Friedman test). eGFR results are provided in Figure 1 to the left, demonstrating that the CKD-EPI formula yielded the highest and the U25 formula yielded the lowest results. While the U25 and modified Schwartz results looked similar, the non-parametric Wilcoxon test revealed a statistical difference of 6.3 mL/min/1.73 m^2^ (*p* < 0.0001).

### 3.3. Correlation Analysis

**Figure 2:**
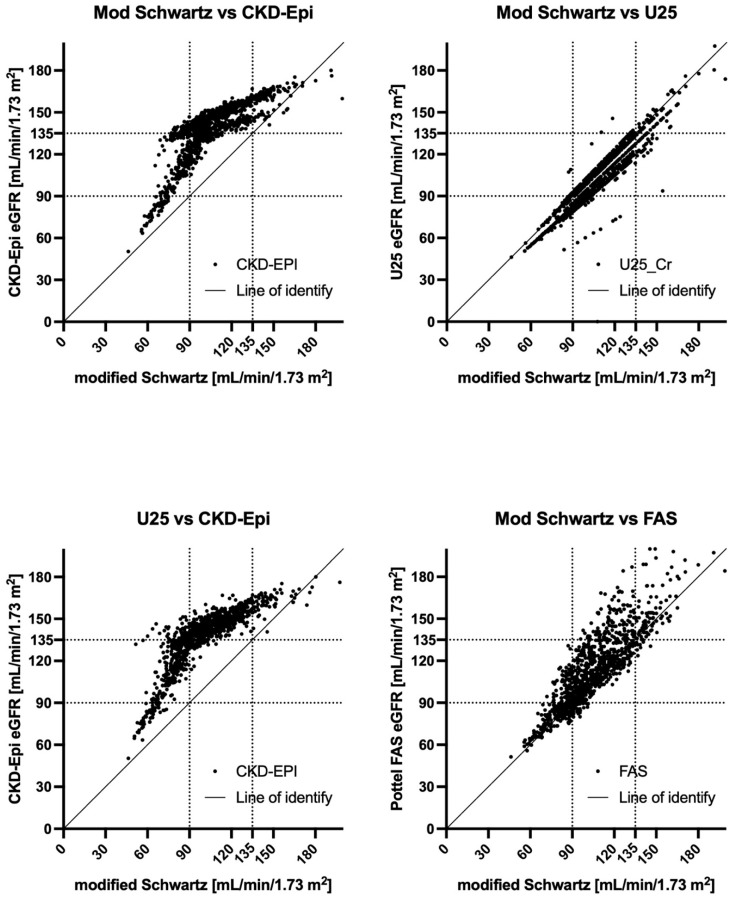
The relationship of the eGFRs with each other. CKD-EPI—Inker’s eGFR formula for adults [13]; U25—Pierce’s CKiD eGFR for patients aged 2–25 years of age [11]; modified Schwartz—Schwartz’ updated formula of the bedside eGFR for enzymatic creatinine measurements [10]; FAS—Pottel’s full age spectrum [19].

**Figure 3:**
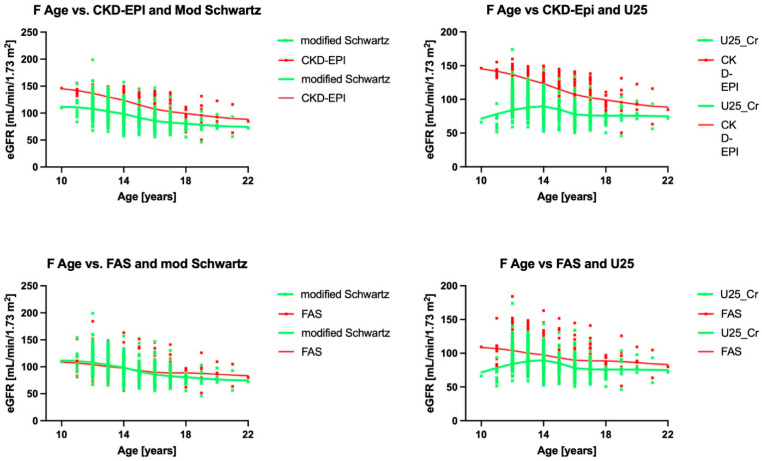
The relationship of the eGFRs with each other by age for females only. The smoothed splines only converged between FAS and modified Schwartz in female participants under 15. CKD-EPI—Inker’s eGFR formula for adults [13]; U25—Pierce’s CKiD eGFR for patients aged 2–25 years of age [11]; modified Schwartz—Schwartz’ updated formula of the bedside eGFR for enzymatic creatinine measurements [10]; FAS—Pottel’s full age spectrum [19].

All eGFR results correlated with each other. The best correlation was between the U25 and modified Schwartz formulas. While all formulas correlated with U25, the patterns were somewhat different, as shown in the scatter plots in Figure 2. CKD-EPI and the modified FAS formula yielded bimodal distributions. We, therefore, performed a subgroup analysis for males and females, finding that agreement was worse in females (Figure 3).

### 3.4. Analysis by Age

**Figure 4:**
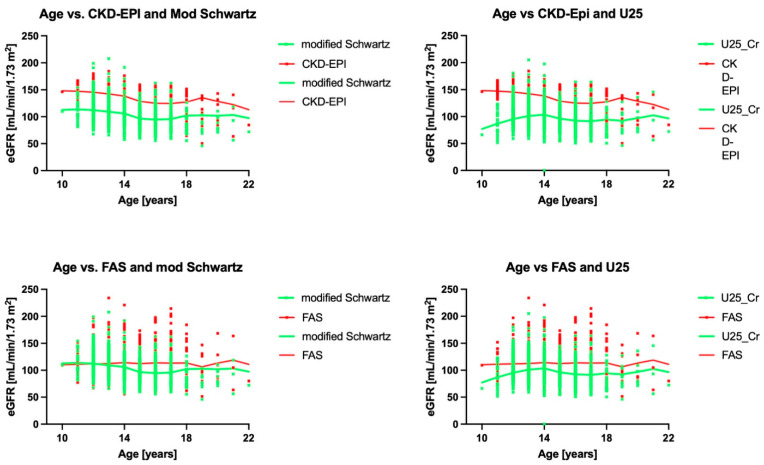
The relationship of the eGFRs with each other by age and smoothed spline average lines for both sexes. The smoothed splines only converged between FAS and modified Schwartz in participants under 14 years. CKD-EPI—Inker’s eGFR formula for adults [13]; U25—Pierce’s CKiD eGFR for patients aged 2–25 years of age [11]; modified Schwartz—Schwartz’ updated formula of the bedside eGFR for enzymatic creatinine measurements [10]; FAS—Pottel’s full age spectrum [19].

Figure 4 summarizes stratified analysis by age. Of interest is that the comparison of U25 and modified Schwartz versus CKD EPI showed inflection points at a CKD-EPI average of 135 mL/min/1.73 m^2^, whereas in the other two formulae, it was at 90 mL/min/1.73 m^2^.

### 3.5. Classification of CKD Stages

The modified Schwartz identified 272 (25.6%) and 9 (0.8%) measurements as having CKD 2 and 3, respectively; the U25 formula identified 376 (35.3%) and 25 (2.4%) measurements as having CKD 2 and 3, respectively; the FAS formula identified 218 (20.5%) and 49 (4.6%) measurements as has having CKD 2 and 3, and the modified FAS formula identified 266 (25.0%) and 16 (0.2%), respectively; while the CKD-EPI formula identified significantly fewer, namely 50 (4.7%) and 1 (0.1%) measurements as has having CKD 2 and 3, respectively. These differences were significant (Chi-square 385.4, df = 6, *p* < 0.0001). The U25 identified 660 (62.5%) measurements as having normal eGFR compared to 1010 (95.2%) measurements with the CKD-EPI formula.

### 3.6. Subgroup Analysis for ≥18 Years of Age Only

For the 46 participants ≥18 years of age, their eGFR was normally distributed. The mean results by the different formulas were as follows: modified Schwartz eGFR was 84.9 ± 17.5 mL/min/1.73 m^2^, U25 eGFR was 85.4 ± 23.1 mL/min/1.73 m^2^, FAS eGFR was 85.4 ± 23.1 mL/min/1.73 m^2^, modified FAS eGFR was 90.19.4 ± 22.06 mL/min/1.73 m^2^, and CKD-EPI eGFR was 108.4 ± 26.7 mL/min/1.73 m^2^. The box and whisker plot is given in Figure 1. The mean bias between CKD-EPI and U25 was +23.0 ± 10.9 (95% limits of agreement from 1.70 to 44.2) mL/min/1.73 m^2^.

### 3.7. Subgroup Analysis for Hyperfiltration

The CKD-EPI and the modified Schwartz formula identified 97 measurements with an eGFR >135 mL/min/1.73 m^2^. The proportion of measurements with hyperfiltration using the FAS eGFR was 90, whereas U25 only identified 63 measurements. Median (IQR; range) eGFR using the various formulas include modified Schwartz formula 145.9 (140.9, 154.5, and 136–207.9) mL/min/1.73 m^2^; the FAS formula 151.8 (141.7, 173.6; 123.7–234.1) mL/min/1.73 m^2^; the U25 formula 139.9 (131.4, 150.1; 93.7–205.1) mL/min/1.73 m^2^; and the CKD-EPI formula 162.5 (158.4, 165.9; 141.1–184.4) mL/min/1.73 m^2^. Using ANOVA, the estimates were again significantly different (*p* < 0.0001, Friedman statistics 154.1). The U25 estimated eGFR was considerably lower than the modified Schwartz eGFR (*p* < 0.0001, Wilcoxon matched-pairs signed rank test).

### 3.8. Subgroup Analysis of Longitudinal eGFR Measurements

**Figure 5:**
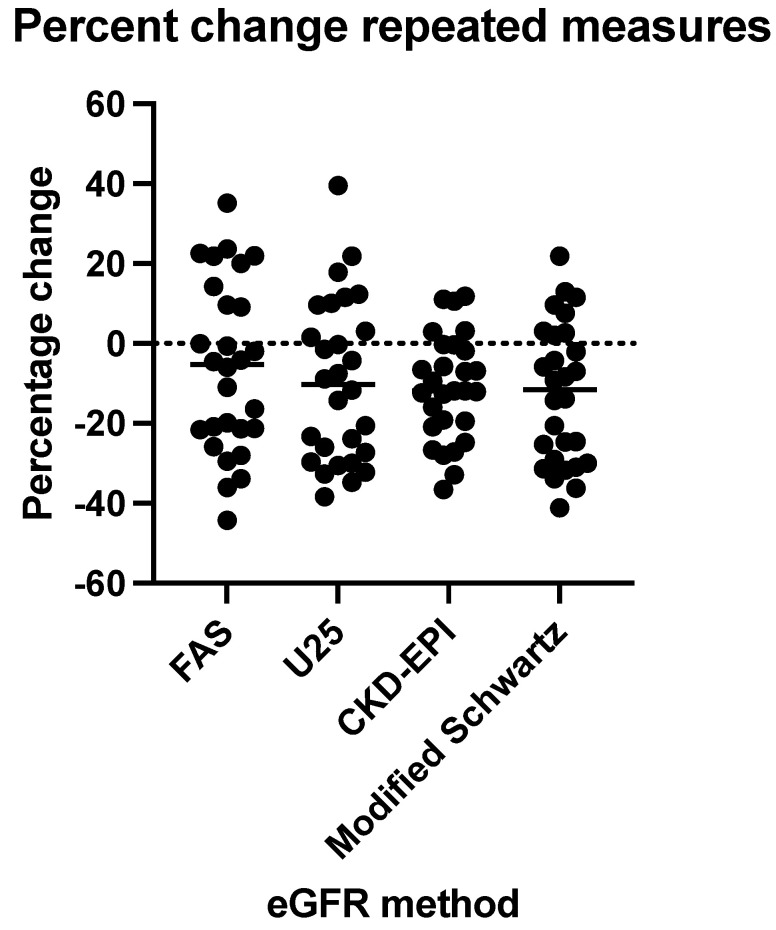
Percentage change in eGFR for the four methods. CKD-EPI—Inker’s eGFR formula for adults [13]; U25—Pierce’s CKiD eGFR for patients aged 2–25 years of age [11]; modified Schwartz—Schwartz’ updated formula of the bedside eGFR for enzymatic creatinine measurements [10]; FAS—Pottel’s full age spectrum [19].

We investigated if this difference among the various eGFR formulae improved with age. Twenty-eight participants had two consecutive measurements with a median of 2 years (IQR 2,2). FAS eGFR dropped on average by −6.03 ± 21.46%, U25 by −9.64 ± 20.36%, CKD-EPI by −11.1 ± 13.12% and modified Schwartz by −12.61 ± 17.50% (Figure 5). Using Geisser–Greenhouse correction for ANOVA, the matching was effective (Chi-square 115.5, df 1, *p* < 0.0001), and the percentage drop was statistically significant (*p* = 0.0206, Geisser–Greenhouse epsilon 0.6750). As such, all five eGFR models behaved similarly.

We then analyzed the classifications using the CKD stage. The results of the four formulae are summarized in Table 3. Measurements were significantly different using the Chi-square test, with the first measurement showing (*p* = 0.0325, chi-square 8.768, df = 3) and the second measurement showing (*p* = 0.0003, chi-square 18.53.88, df = 3), suggesting that CKD-EPI would classify the eGFR of many participants as >90 mL/min/1.73 m^2^.

## 4. Discussion

In this large community-based AYA population that has been traditionally under-investigated, at risk for CKD, and needing targeted screening for impaired GFR, we found poor agreement between all four eGFR formulae at baseline, with some data from longitudinal samples. There were differences among all five eGFR formulae, with the lowest CKD identification with CKD-EPI, and the highest identification with the U25 formula. Especially in the normal GFR range, CKD-EPI results were substantially higher than the modified Schwartz and U25 estimates. The two formulae developed for the entire age spectrum (FAS and modified FAS) behaved more like the modified Schwartz and U25 estimates. While significantly different, the best agreement was between the modified Schwartz and the U25 formulae. Given the similar ranges of the other 3 GFR estimates, this raises doubts about the usability of CKD-EPI for patients aged 10–23. Admittedly, guidelines only suggest using it from age 18 onwards, but a few publications suggested its use in adolescents, either alone [21] or as the average of both [22]. Furthermore, our subgroup analysis demonstrated that for a median follow-up of 2 years (range 1–5 years), the significant difference in the classification of CKD stage did not improve. Our data do not support using CKD-EPI for this population, while the two FAS formulae designed for the entire age group may be most appropriate. With a gold standard measured GFR, we could determine which eGFR formula has the best accuracy and precision. However, our data suggest that CKD-EPI overestimates the GFR. Furthermore, measuring GFR in such a population, for instance, with iohexol or 51Cr EDTA, is not feasible.

Several groups have identified the problem with switching from pediatric to adult formulae [14,15,16,19]. Pottel reported an increase of 21 mL/min/1.73 m^2^ [2,23]. Schellekens et al. also demonstrated an implausible jump in kidney function upon the transition age of 68 ADPKD patients [24]. A study comparing iohexol-based measured GFR with CKD-EPI and U25 showed the worst bias and accuracy in 53 AYA with type 1 diabetes [25]. Part of the problem is the need for more young adults in the original CKD-EPI cohort [13]. Regression analysis only works in the parameter range where the data were generated, as we have shown for various measured GFR levels [26]. Björk et al. suggested correcting the CKD-EPI equation with age-adjusted creatinine values [27]. Our findings agree with these observations, and switching to CKD-EPI may not be feasible for the everyday screening of patients.

An interesting observation is the inflection point when comparing the CKD-EPI formula with the U25 and modified Schwartz formula. Leslie Inker recently analyzed the diagnostic performance in young adults and described a similar distribution when plotting CKD-EPI 2021 with the U25 formula (top Figure) [28]. The conclusion from that paper was an underestimation of the GFR using U25 in patients with higher mGFR, similar to our own findings [18] and Nyman’s findings [14]. There is growing evidence that the U25 formula using creatinine alone may not be ideal for screening participants such as those who were subject to our field study.

It is also essential to correctly identify patients with hyperfiltration, as they may also be at risk [29]. The CKD-EPI and the modified Schwartz formula identified the same participants; however, U25, which included a dataset with very few hyperfiltration participants, identified a significantly lower number. Unfortunately, hyperfiltration influences serum creatinine, whereas the impact on cystatin C is much less [30].

It is well established that adding cystatin C eGFR and averaging creatinine and cystatin C-based GFR estimates improves the accuracy of estimating GFR [1,11]. We have recently shown that averaging the Filler formula for cystatin C [31], and the modified Schwartz formula [10] for eGFR had a better diagnostic performance than the U25 or the CKD-EPI formula in patients aged 2–20, especially when eGFR was greater than 75 mL/min/1.73 and even hyperfiltration [18]. However, cystatin C is not widely available, and considerable barriers exist to its implementation worldwide [32]. A recent NICE recommendation strongly supported cystatin C use but highlighted the knowledge gap among young adults [33]. In our present study, we did not have serum cystatin C concentrations. When combining cystatin C and creatinine, there are problems among AYA with U25 in the higher eGFR range [14].

Our data suggest that we should adopt pediatric eGFR formulae in young adults, though it remains to be seen if the time of convergence with CKD-EPI is 25, 30, or even 40 years of age. Our study clearly describes the magnitude of the potential underestimation of CKD when using the CKD-EPI formula in community-based children and AYA. This is particularly important if we have a GFR estimate during childhood and want to assess the evolution of the patient during the transition to adult-focused care.

Our study identified participants with potentially undetected CKD stages 2 and 3. People in this region are at risk of KDu or Mesoamerican nephropathy, a poorly understood entity with a higher incidence in AYA males. This nephropathy may be related to crop/agrochemical/heat/heavy metal exposures, dehydration, high fructose rehydration, genetic predisposition, or infections that may start in childhood [9]. Targeted screening of individuals at risk for CKD is paramount.

Limitations of this study include that we did not have a gold standard measured GFR method, although one could argue that the modified Schwartz formula would have the best accuracy compared to ^99m^Tc DTPA measured GFR [18]. However, the modified Schwartz and U25 formulae were derived using iohexol clearance. We cannot comment on accuracy and bias against measured GFR. The data are limited to a homogenous Mexican population from a single Meso-American region. The slight predominance of female participants may have introduced a potential bias. We did not have simultaneous cystatin C concentrations nor data on muscle mass in these participants. The analysis of the longitudinal data was underpowered. Some participants were as tall as 183 cm, which renders the calculation of body surface area inaccurate, given the worldwide increase in height.

## 5. Conclusions

In this Mexican community-based study in an area at risk for CKD, we demonstrated very different proportions of participants classified as having an eGFR < 90 mL/min/1.73 m^2^ based on which eGFR method was used. Based on our findings, switching from a pediatric approach formula to CKD-EPI may not be accurate, regardless of whether the entire age range of our study was analyzed or the subgroup of over 17-year-old participants. By contrast, the FAS and the modified FAS formulae yielded similar results as modified Schwartz and U25. The spread of the modified FAS formula was the lowest, and since the formula is designed for all age groups, it may currently be the best approach.

It is important to recognize that no single equation based on serum creatinine can be universally valid across all age groups, muscle mass variations, or GFR levels measured by gold-standard clearance methods. Serum creatinine-based GFR estimates are inherently imprecise due to multiple factors, including variations in muscle mass, dietary influences, drug effects on creatinine metabolism, and the presence of hyperfiltration. Moreover, while serum creatinine and GFR are inversely related, their relationship is hyperbolic—meaning that at high GFR levels, small fluctuations in serum creatinine may not significantly impact eGFR estimates, whereas in low GFR states, even minor changes in serum creatinine can lead to substantial variations in eGFR. Therefore, clinicians should interpret eGFR values cautiously, particularly in populations at risk for misclassification, such as adolescents and young adults transitioning between pediatric and adult estimation methods.

Moreover, we urgently need studies that pool existing data with a gold standard measured GFR and simultaneous enzymatic creatinine and cystatin C that is measured against internationally certified reference labs from various regions of the world to answer the question of how best to estimate GFR in adolescents and young adults, especially with a measured GFR of >75 mL/min/1.73 m^2^. The issue of whether absolute or ideal weight should be used for the gold standard GFR must be resolved, and potentially, all formulae must be recalculated.

## Figures and Tables

**Table 1:** Characteristics and kidney parameters of the study participants.

Characteristics	n = 914
Age, years (median, IQR)	13 (12–15)
Female	503 (55.0)
Male	411 (45.0)
Anthropometry	
Height cm (median, IQR)	158 (152, 160)
BMI, n (%)	
Low weight	20 (2.2)
Normal weight	608 (66.5)
Overweight	171 (18.7)
Obesity	115 (12.6)
BMI kg/m^2^, (median, IQR)	21.0 (18.9–24.2)
WHtR, (median, IQR)	0.47 (0.44–0.52)
High blood pressure, n (%)	82 (8.97)
Premature birth <36 weeks gestational, n (%)	110 (12.04)
Poverty, n (%)	
No	392 (42.89)
Moderate	458 (50.11)
Extreme	64 (7)
Kidney parameters	
Serum creatinine mg/dL (median, IQR)	0.62 (0.53–0.72)
ACR mg/g, (median, IQR)	4.94 (<LOD–22.53)
ACR mg/g, n (%)	
<30	741 (81–07)
30–300	169 (18.49)
>300	4 (0.44)

Abbreviations: BMI, body mass index; WHtR, waist height to ratio; ACR, albumin creatinine ratio; LOD, limit of detection; IQR, interquartile range.

**Table 2:** Descriptive statistics of the four eGFR calculations.

	CKD-EPI [mL/min/1.73 m^2^]	U25_Cr [mL/min/1.73 m^2^]	Modified Schwartz [mL/min/1.73 m^2^]	FAS [mL/min/1.73 m^2^]
Number of values	1061	1061	1061	1061
Minimum	50.4	46.3	46.2	51.4
25% Percentile	129.9	83.35	89.1	92.85
Median	140.8	96.9	101.3	110.2
75% Percentile	149.3	113.2	118.6	129.9
Maximum	184.4	205.2	207.9	234.1
Range	134	158.9	161.7	182.7
Mean	136.2	99.34	104.7	113
Std. Deviation	20.58	22.7	22.3	26.82
Std. Error of Mean	0.6318	0.6969	0.6847	0.8233
Lower 95% CI of geo. mean	133	95.5	101.2	108.4
Upper 95% CI of geo. mean	135.8	98.15	103.8	111.5
Skewness	−1.179	0.6179	0.6355	0.6794
Kurtosis	1.356	0.7329	0.7623	0.7172

Abbreviations: FAS, Full Age Spectrum; Std, standard; geo, geometric.

**Table 3:** Classification of CKD on longitudinal follow-up in 28 patients. The number of patients with an eGFR < 90 mL/min/1.73 m^2^ significantly differed among the four methods and did not improve after a medium of two years of follow-up.

First Measurement			Second Measurement	
	CKD Stage 1	CKD Stage 2+	CKD Stage 1	CKD Stage 2+
Modified Schwartz	14	14	7	21
U25	12	17	6	22
FAS	15	13	10	18
CKD-EPI	22	6	20	8

## Data Availability

The original contributions presented in this study are included in the article. Further inquiries can be directed to the corresponding authors.
